# Severe complications following transarterial microembolization for a micro arterio-venous fistula in a patient with chronic venous ulcer: a case report

**DOI:** 10.3389/fradi.2025.1613940

**Published:** 2025-07-18

**Authors:** Wankarn Boonlorm, Panat Nisityotakul

**Affiliations:** Department of Radiology, Vachira Phuket Hospital, Phuket, Thailand

**Keywords:** transarterial microembolization, imipenem/cilastatin, chronic venous ulcers, musculoskeletal pain, embolization complications, patient safety

## Abstract

Transarterial microembolization (TAME) has gained recognition as a minimally invasive treatment for chronic musculoskeletal pain, demonstrating significant efficacy with a favorable safety profile ( 1, 2). However, complications remain underreported. This case report describes the first documented severe adverse event in a patient with a chronic venous ulcer undergoing TAME for a micro arteriovenous fistula (AVF). The patient developed significant complications, including extensive leg swelling, skin changes, and cellulitis requiring prolonged inpatient care. These findings highlight the importance of patient selection and embolic agent considerations to mitigate potential risks associated with TAME.

## Introduction

TAME is an emerging interventional technique for chronic musculoskeletal pain and vascular anomalies, with reported success in osteoarthritis and pain syndromes (2, 3). While previous studies have reported minimal complications, there is limited data on its safety in patients with vascular compromises. This case highlights the first documented severe complication following TAME with Imipenem/Cilastatin (IPM/CS), raising concerns about embolic agent selection and patient eligibility.

## Case presentation

**Figure 1:**
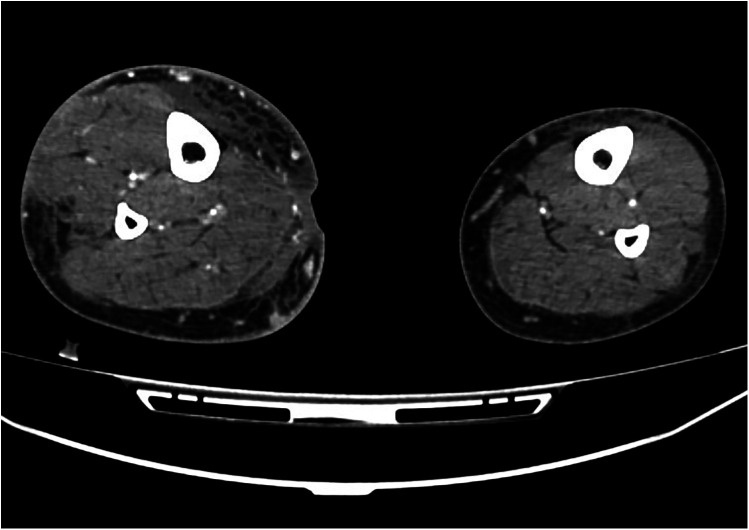
Computed tomography angiography revealed a micro arteriovenous fistula (AVF) in the right leg.

**Figure 2:**
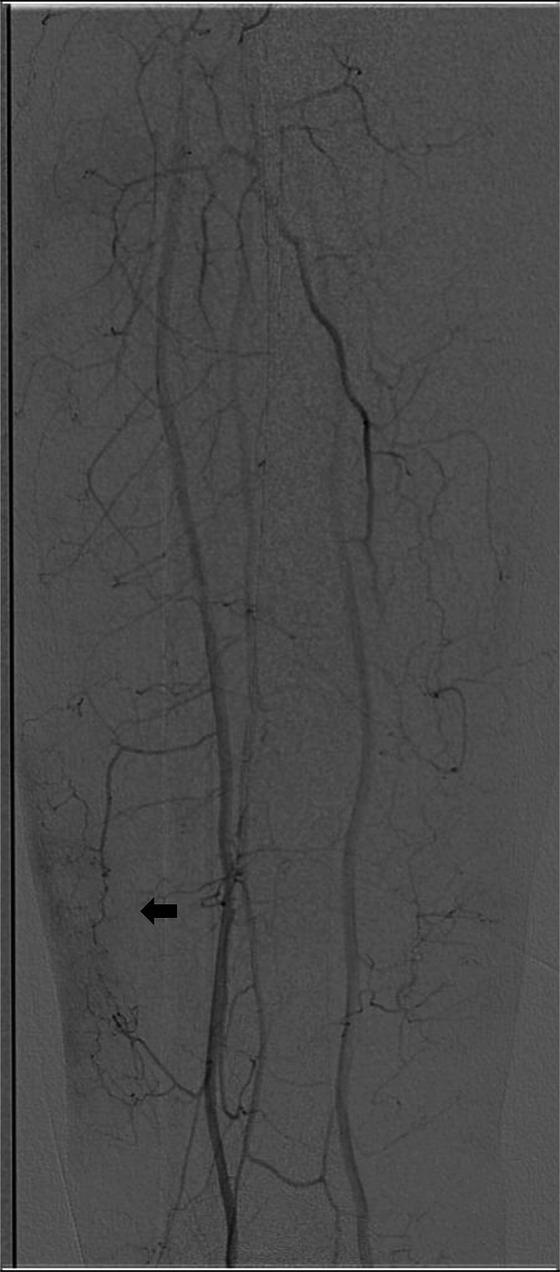
Digital subtraction angiography (DSA) confirmed the presence of abnormal vascular blush (arrow 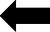).

A 65-year-old male with a history of chronic venous ulcer presented with persistent leg pain and swelling. Clinical examination revealed an ulcerated lesion over the lower leg with surrounding skin hyperpigmentation and signs of chronic venous insufficiency. Computed tomography angiography identified a micro AVF in the right leg (Figure 1), confirmed by digital subtraction angiography (DSA) (Figure 2). Given his refractory pain and non-healing ulcer, he was referred for TAME as an alternative to surgical management.

## Preprocedural assessment

Baseline laboratory investigations showed normal coagulation parameters and no active infection. Imaging confirmed a high-flow micro AVF supplying the ulcerated region. After multidisciplinary discussion, TAME was planned to use intra-arterial IPM/CS, based on prior studies supporting its efficacy in osteoarthritis and neovascularization (3).

## Procedure

A 4-French sheath was introduced via femoral artery access under ultrasound guidance. Superselective catheterization of the target feeding arteries was achieved using a microcatheter. A slow intra-arterial infusion of IPM/CS was performed, with a total of 1.5 ml of IPM/CS (500 mg in 5 ml contrast) administered into the anterior tibial artery under fluoroscopic guidance. Post-embolization angiography confirmed the occlusion of the abnormal microvasculature.

## Postprocedural course

**Figure 3:**
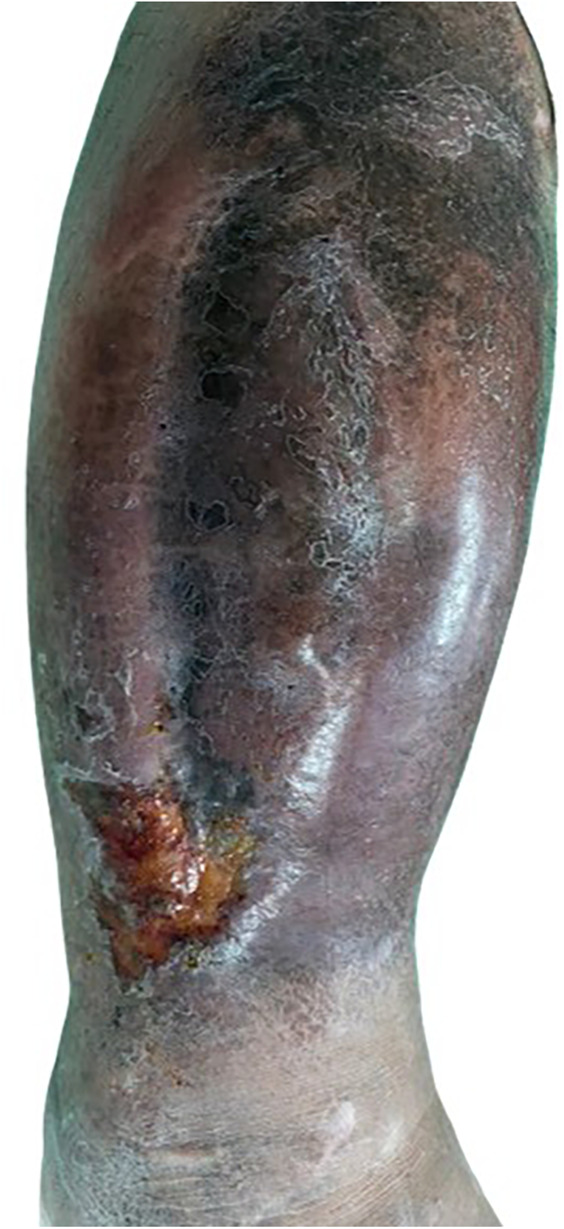
Two days post-TAME, the patient developed worsening leg swelling, erythema, and multiple fluid-filled blebs.

**Figure 4:**
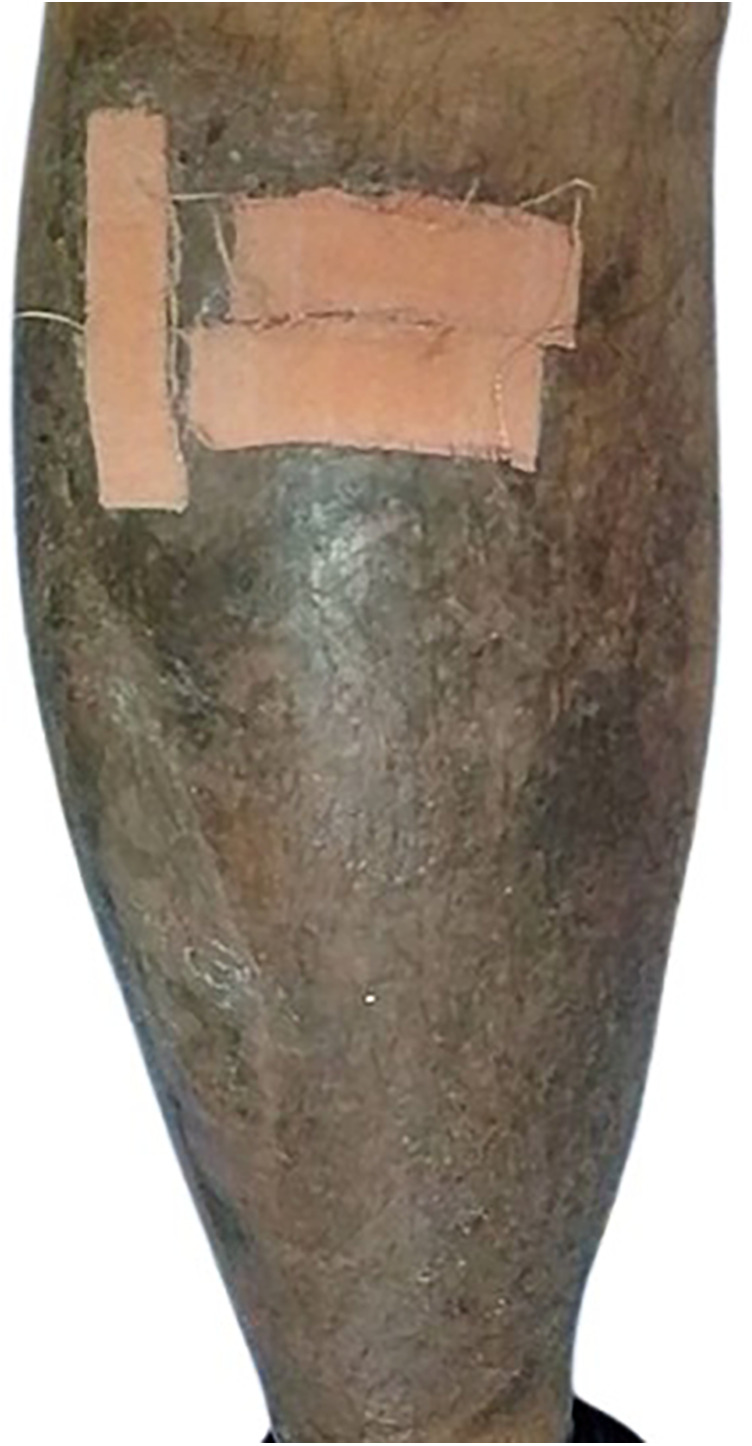
The ulcer healing was significantly delayed despite conservative treatment.

The patient was discharged without immediate complications. However, two days later, he returned with worsening lower limb swelling, erythema, and multiple fluid-filled blebs (Figure 3). These symptoms suggested acute tissue ischemia and an inflammatory response, raising concern for severe non-target embolization. The patients developed cellulitis and worsening tissue breakdown necessitated prolonged inpatient care, including intravenous antibiotics, wound debridement, and limb elevation. Over subsequent weeks, the patient gradually improved with conservative management, though ulcer healing was significantly delayed (Figure 4).

## Discussion

TAME has been extensively studied for osteoarthritis and chronic pain syndromes (1, 3), yet its use in patients with chronic venous ulcers and vascular compromise remains controversial. Liang et al. (4) reported the safety of IPM/CS infusion in treating hand osteoarthritis, noting only transient swelling and discoloration. Similarly, Kubo et al. (5) found high technical success with minimal complications in a cohort of 92 patients undergoing intra-arterial IPM/CS injection.

However, Lin et al. (6) highlighted potential risks, including arterial dissection and catheter-related complications. Our case presents a novel and severe complication, likely resulting from non-target embolization and impaired microvascular perfusion in an ulcerated limb. The inflammatory response following embolization may have exacerbated tissue hypoxia, leading to severe skin breakdown and prolonged recovery.

This case underscores the importance of careful patient selection, particularly for individuals with preexisting vascular insufficiency. The choice of embolic agent remains critical; while IPM/CS has demonstrated efficacy, its effects on compromised microcirculation warrant further investigation.

## Conclusion

This case report highlights a rare but significant complication following TAME in a patient with chronic venous ulcer. While TAME remains a promising technique for chronic pain management, its application in patients with vascular compromise should be approached with caution. Future studies should focus on optimizing embolic agent selection and refining patient eligibility criteria to mitigate risks.

## Data Availability

The datasets presented in this study can be found in online repositories. The names of the repository/repositories and accession number(s) can be found in the article/Supplementary Material.
